# Combination of GLP-1 Receptor Activation and Glucagon Blockage Promotes Pancreatic *β*-Cell Regeneration *In Situ* in Type 1 Diabetic Mice

**DOI:** 10.1155/2021/7765623

**Published:** 2021-11-25

**Authors:** Liangbiao Gu, Dandan Wang, Xiaona Cui, Tianjiao Wei, Kun Yang, Jin Yang, Rui Wei, Tianpei Hong

**Affiliations:** Department of Endocrinology and Metabolism, Peking University Third Hospital, Beijing 100191, China

## Abstract

Pancreatic *β*-cell neogenesis *in vivo* holds great promise for cell replacement therapy in diabetic patients, and discovering the relevant clinical therapeutic strategies would push it forward to clinical application. Liraglutide, a widely used antidiabetic glucagon-like peptide-1 (GLP-1) analog, has displayed diverse *β*-cell-protective effects in type 2 diabetic animals. Glucagon receptor (GCGR) monoclonal antibody (mAb), a preclinical agent that blocks glucagon pathway, can promote the recovery of functional *β*-cell mass in type 1 diabetic mice. Here, we conducted a 4-week treatment of the two drugs alone or in combination in type 1 diabetic mice. Although liraglutide neither lowered the blood glucose level nor increased the plasma insulin level, the immunostaining showed that liraglutide expanded *β*-cell mass through self-replication, differentiation from precursor cells, and transdifferentiation from pancreatic *α* cells to *β*-cells. The pancreatic *β*-cell mass increased more significantly after GCGR mAb treatment, while the combination group did not further increase the pancreatic *β*-cell area. However, compared with the GCGR mAb group, the combined treatment reduced the plasma glucagon level and increased the proportion of *β*-cells/*α*-cells. Our study evaluated the effects of liraglutide, GCGR mAb monotherapy, and combined strategy in glucose control and islet *β*-cell regeneration and provided useful clues for the future clinical application in type 1 diabetes.

## 1. Introduction

Pancreatic *β*-cell dysfunction and cell mass loss are a pivotal pathogenesis in both type 1 diabetes (T1D) and type 2 diabetes (T2D) [1, 2]. Therefore, it is highly necessary to preserve *β*-cell function and expand *β*-cell mass for diabetes treatment. Glucagon-like peptide-1- (GLP-1-) based therapies, including GLP-1 receptor agonists and dipeptidyl peptidase-4 inhibitors, have several beneficial effects on pancreatic *β*-cells, including upregulating insulin gene transcription and biosynthesis, potentiating glucose-stimulated insulin secretion, and promoting *β*-cell regeneration by promoting *β*-cell proliferation, inhibiting *β*-cell apoptosis, and inducing stem cells to differentiate into *β*-cells [3, 4]. Recent researches have proven that *GLP-1* overexpression or GLP-1 receptor agonists promoted *β*-cell regeneration *via α*- to *β*-cell transdifferentiation [5–7]. Although GLP-1-based therapy shows various beneficial effects in T2D animals and humans, these drugs cannot be used alone for T1D treatment [8]. Whether GLP-1-based therapy has similar effects on *β*-cell protection, especially for *β*-cell regeneration in T1D, needs to be determined, and which therapy should be combined with GLP-1 should be evaluated.

REMD2.59, a fully competitive antagonistic glucagon receptor (GCGR) monoclonal antibody (mAb), has a strong hypoglycemic effect in T1D and T2D rodents and nonhuman primates [9–11]. A randomized clinical trial showed that REMD477, another GCGR mAb that has an affinity for the GCGR equivalent to that of REMD2.59, improved glycemic control in patients with T1D without serious adverse effects [12]. Our previous findings suggested that treatment with the GCGR mAb increased the *β*-cell mass by promoting *α*- to *β*-cell conversion [13]. However, GCGR mAb substantially increased pancreatic *α*-cell mass and plasma glucagon levels. Notably, GLP-1 receptor agonists have the ability of inhibiting glucagon secretion. When combined with GCGR mAb, the GLP-1 receptor may avoid hyperglucagonemia.

In this study, we investigated the possible effect of liraglutide, a commonly used GLP-1 receptor agonist, on *β*-cell regeneration in T1D mice, and evaluated the combined effect of liraglutide with GCGR mAb. Our study provides new clues for the clinical therapy to maintain glucose homeostasis and promote pancreatic *β*-cell neogenesis in T1D patients.

## 2. Materials and Methods

### 2.1. Animals and Intervention

All the animal experiments were conducted at Peking University Health Science Center (Beijing, China) and approved by the Institutional Animal Care and Use Committee. B6.Cg-Tg(Gcg-cre)1Herr/Mmnc (cre expression in pancreatic *α*-cell lineage) and B6.Rosa26-LSL-Cas9-tdTomato/J (when crossed to a cre recombinase-expressing strain, red fluorescence protein (RFP) expression is observed in the cre-expressing tissues) mice were crossed to generate pancreatic *α*-cell lineage-tracing mice, namely, glucagonCre-RFP mice. Eight-week-old male glucagonCre-RFP mice or C57BL/6J mice (Vital River Animal Center, Beijing, China) were injected intraperitoneally with streptozotocin (STZ, 150 mg/kg; Sigma-Aldrich, Saint Louis, MO) in citric acid buffer (0.1 mol/L, pH 4.2) to establish a T1D model. The diabetic condition was confirmed if fasting blood glucose was 11.1 mmol/L or the random blood glucose was 16.7 mmol/L at least twice. If the blood glucose level was higher than 33.3 mmol/L (the upper detection limit of the blood glucose meter), 33.3 mmol/L was recorded. The diabetic C57BL/6J animals were treated for four weeks by intraperitoneal administration of liraglutide (0.2 mg/kg, twice daily; Novo Nordish, Denmark), REMD2.59 (a human GCGR mAb, 5 mg/kg, once a week; REMD Biotherapeutics, Camarillo, CA, USA), a combination of two agents, or saline (as control). There were 6-8 mice in each group. The diabetic glucagonCre-RFP mice were treated for four weeks by intraperitoneal administration of liraglutide or saline. Mice were fasted 8 h for the measurement of fasting blood glucose by the glucose oxidase method. After the four-week treatment, the mice were sacrificed, and then, plasma and pancreas were collected. Specific ELISA kits were used for detecting insulin (Millipore, Saint Charles, MO, USA) and glucagon (R&D System, Minneapolis, MN, USA) according to the manufacturer's instructions.

### 2.2. Immunofluorescence Staining

Pancreases were fixed with 10% formalin at 4°C overnight and embedded in paraffin, and 5 *μ*m thick sections were prepared. The sections were incubated with primary antibodies at 4°C overnight and secondary antibodies for 1 h at room temperature, followed by washing and staining with DAPI (1 *μ*g/mL, Sigma-Aldrich). Images were captured under a confocal fluorescence microscope (Leica TCS SP8; Leica Microsystems Inc., Wetzlar, Germany) or an automatic digital slide scanner (Panoramic MIDI, 3D HISTECH, Budapest, Hungary). For cell quantification, 6 to 9 equally spaced sections (which covered the entire pancreas) per pancreas were imaged, and the total numbers of positive staining cells from 6 mice per group were counted by using ImageJ software.

The following antibodies and dilutions were used: rabbit antiglucagon (1 : 800, Cell Signaling Technology, Beverly, MA), mouse antiglucagon (1 : 400, Sigma-Aldrich), mouse anti-insulin (1 : 800, Sigma-Aldrich), guinea pig anti-insulin (1 : 200, Abcam), mouse antiproliferating cell nuclear antigen (PCNA; 1 : 400, Cell Signaling Technology), rabbit anticytokeratin 19 (CK19; 1 : 400, Abcam), rabbit polyclonal antipancreatic and duodenal homeobox 1 (Pdx1, 1 : 200, Abcam), rabbit monoclonal anti-NK6 homeobox 1 (Nkx6.1, 1 : 400 , Abcam), rabbit polyclonal antiproprotein convertase 1/3 (PC1/3, 1 : 400, Millipore), rabbit polyclonal anti-RFP (1 : 200; Abcam), Alexa Fluor 594-conjugated AffiniPure goat antimouse IgG (H+L) (1 : 800, Jackson ImmunoResearch Laboratories, West Grove, PA), Alexa Fluor 488 or 594-conjugated AffiniPure goat antirabbit IgG (H+L) (1 : 800, Jackson ImmunoResearch Laboratories), and Alexa Fluor 647-conjugated AffiniPure goat antiguinea pig IgG (H+L) (1 : 800, Jackson ImmunoResearch Laboratories).

### 2.3. Primary Islets and Islet Cell Line Experiments

Primary islets were isolated from C57BL/6J mice as previously reported [14, 15]. Briefly, the pancreas was perfused by collagenase V (Sigma-Aldrich), and individual islets were handpicked and cultured in the RPMI 1640 medium (Invitrogen, Carlsbad, CA, USA) supplemented with 10% fetal bovine serum (Hyclone, Logan, UT, USA), 2 mmol/L GlutMax, 1 mmol/L sodium pyruvate, and 1% penicillin-streptomycin (Thermo Fisher Scientific, Waltham, MA, USA). Mouse pancreatic *α*-cell line *α*TC1 clone 7 (*α*TC1.9) cells (ATCC, USA) were cultured in Dulbecco's modified Eagle medium (5.5 mmol/L glucose; Invitrogen) supplemented with 10% fetal bovine serum, 15 mmol/L HEPES, 0.1 mmol/L nonessential amino acids, 0.02% bovine serum albumin, 2 mmol/L GlutMax, and 1% penicillin-streptomycin. Primary islets or *α*TC1.9 cells were treated with GCGR mAb (100 nmol/L) or IgG for 24 h, and RNA was collected for further analysis.

### 2.4. Quantitative RT-PCR

Total RNA was extracted with TRIzol reagent (Thermo Fisher Scientific) and reversely transcribed to cDNA using a RevertAid First Strand cDNA Synthesis kit (Fermentas, Vilnius, Lithuania). The quantitative RT-PCR was performed using iQ SYBR Green supermix (BioRad Laboratories, Hercules, CA, USA) on a QuantStudio 5 Real-Time PCR System (Thermo Fisher Scientific). All experiments were performed in triplicate. Relative quantification for gene expression was calculated using the 2^−*ΔΔ*CT^ method. The primers were synthesized by the Beijing AuGCT DNA-SYN Biotechnology Company (Beijing, China). The primer sequences were as follows: *Glp-1r*: forward: AGCACTGTCCGTCTTCATCA, reverse: AGAAGGCCAGCAGTGTGTAT; *Gapdh*: forward: TGCACCACCACCAACTGCTTAGC, reverse: GGCATGGACTGTGGTCATGAG.

### 2.5. Statistical Analysis

Data are expressed as the mean ± S.E.M. The difference between two groups was analyzed by ANOVA followed by the *post hoc* Tukey-Kramer test or Student's *t-*test (two-tailed) when appropriate. A *p* value < 0.05 was considered statistically significant. Statistical analysis was performed using GraphPad Prism 8.0 (GraphPad Software Inc., San Diego, CA).

## 3. Results

### 3.1. Liraglutide and GCGR mAb Treatments Improve Diabetic Phenotype in T1D Mice

**Figure 1:**
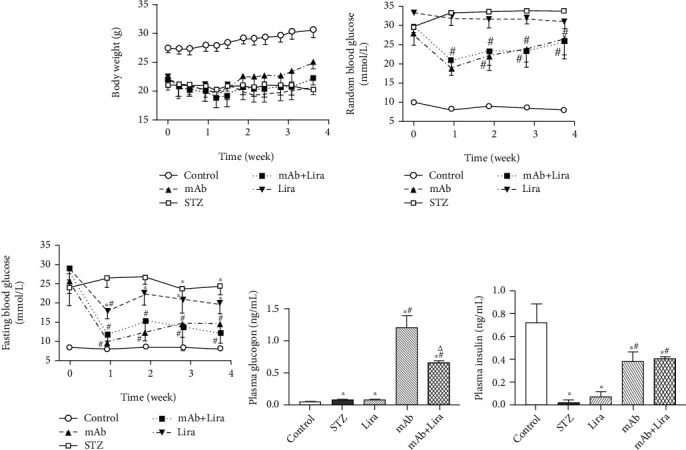
Body weight, blood glucose, plasma insulin, and glucagon levels in T1D and control mice treated with liraglutide, GCGR mAb, or both for four weeks. Eight-week-old male C57BL/6J mice were injected intraperitoneally with 150 mg/kg streptozotocin (STZ) to establish a T1D model. Diabetic mice were treated with liraglutide (Lira, 200 *μ*g/kg), GCGR mAb (5 mg/kg), or liraglutide combined with GCGR mAb. The littermate C57BL/6J mice were used as normal control: (a) body weight; (b) random blood glucose; (c) fasting blood glucose; (d) plasma glucagon; (e) plasma insulin. Data are presented as the mean ± SEM (*n* = 6–8 mice per group). Statistical analysis was conducted by ANOVA. ^∗^*p* < 0.05 vs. control group; ^#^*p* < 0.05 vs. STZ group; ^△^*p* < 0.05 vs. GCGR mAb group.

Compared with the normal control group, the body weight of the T1D mice displayed a significant decrease while the blood glucose level showed obvious increment (Figures 1(a)–1(c)). Neither liraglutide nor GCGR mAb had any effects on the body weights (Figure 1(a)). Liraglutide alone did not decrease the random blood glucose (Figure 1(b)), while displaying a trend to lower fasting blood glucose (*p* = 0.17, Figure 1(c)). The GCGR mAb significantly reduced the random and fasting blood glucose levels (Figures 1(b) and 1(c)). However, the combination of GCGR mAb and liraglutide (mAb+Lira) did not decrease the blood glucose levels further compared to the GCGR mAb group (Figures 1(b) and 1(c)).

After STZ treatment, the plasma insulin level was significantly lower, and the plasma glucagon level was higher in the STZ group than in the control group. Liraglutide did not affect the insulin level or glucagon level (Figures 1(d) and 1(e)). The GCGR mAb treatment significantly upregulated the plasma insulin level and glucagon level when compared with vehicle treatment in T1D mice (Figures 1(d) and 1(e)). The mAb+Lira combination also significantly upregulated the plasma insulin level when compared with the STZ group, but did not show any difference when compared with mAb treatment alone (Figure 1(d)). Notably, although the glucagon level in the combination group was higher than that in the STZ group, it was significantly lower when compared with that in the GCGR mAb group (Figure 1(d)).

### 3.2. Liraglutide and GCGR mAb Treatments Increase Pancreatic *β*-Cell Area of T1D Mice in Varying Degrees

**Figure 2:**
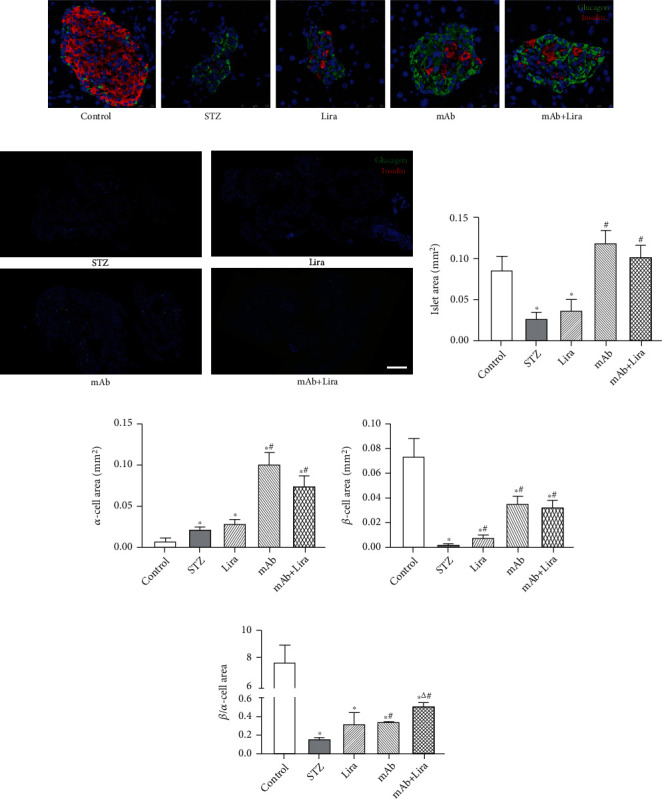
Pancreatic histological analysis of pancreatic *α*-cell and *β*-cell mass in T1D and control mice treated with liraglutide, GCGR mAb, or both for four weeks. (a) Representative images of islets immunostained for glucagon and insulin. Scale bar = 50 *μ*m. (b) Representative images of islets immunostained for DAPI, glucagon and insulin in panoramic view. Scale bar = 2000 *μ*m. (c–f) Total islet area, *α*-cell area, *β*-cell area per pancreatic slice, and the ratio of *β*-cell area to *α*-cell area. *n* = 6 − 9 sections/mouse multiplied by 6 mice/group. Data are expressed as the mean ± SEM. Statistical analysis was conducted by ANOVA. ^∗^*p* < 0.05 vs. control group; ^#^*p* < 0.05 vs. STZ group; ^△^*p* < 0.05 vs. GCGR mAb group.

Histological analysis of the pancreatic islets was carried out by using double-labeled immunofluorescence staining. STZ significantly decreased the entire islet area, with the level less than 1/3 of the normal control (Figures 2(a)–2(c), Table S1). STZ strikingly decreased *β*-cell area and increased *α*-cell mass compared with the normal control (Figures 2(d) and 2(e), Table S1). Liraglutide treatment did not change the total islet area and *α*-cell area, but liraglutide increased the *β*-cell area, thus having a tendency to increase the *β*/*α*-cell area proportion when compared with the STZ group (Figures 2(b)–2(f), Table S1). GCGR mAb treatment increased the total islet area, *β*-cell area, *α*-cell area, and *β*/*α*-cell area proportion when compared with the STZ group (Figures 2(c)–2(f), Table S1). Similarly, the mAb+Lira combination also greatly increased the total islet area and *β*-cell area when compared with the STZ group (Figures 2(c) and 2(d), Table S1), but showed no difference with the mAb group. The mAb+Lira combination increased the *α*-cell area when compared with the STZ group, while decreasing the *α*-cell area when compared with the mAb group (Figure 2(e), Table S1). Therefore, the mAb+Lira combination increased the *β*/*α*-cell area proportion significantly when compared with the STZ or mAb group (Figure 2(f), Table S1).

### 3.3. Liraglutide and GCGR mAb Treatments Promote *β*-Cell Self-Replication in T1D Mice

**Figure 3:**
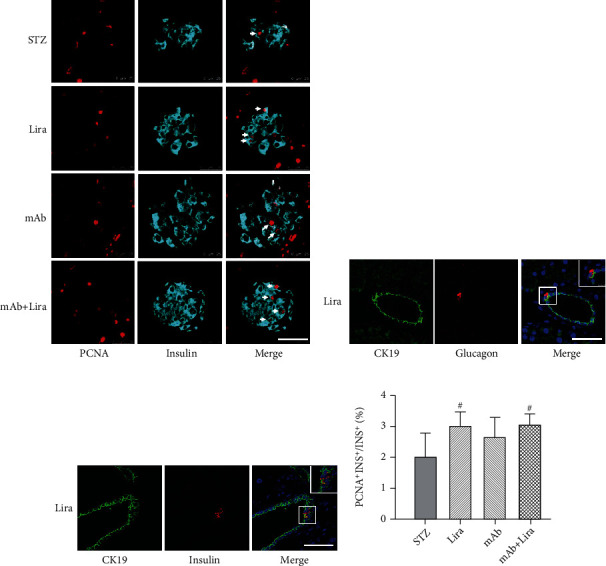
Pancreatic histological analysis of cell proliferation and ductal cell transformation in T1D mice treated with liraglutide, GCGR mAb, or both for four weeks. (a) Representative images of coimmunostaining with insulin and PCNA (proliferating cell nuclear antigen). Scale bar = 50 *μ*m. (b, c) Representative images of coimmunostaining CK19 (cytokeratin 19) with glucagon or insulin. Scale bar = 50 *μ*m. (d) Quantification of the proliferating *β*-cells. *n* = 6 − 8 sections/mouse multiplied by 6 mice/group. Data are expressed as the mean ± SEM. Statistical analysis was conducted by ANOVA. ^#^*p* < 0.05 vs. STZ group. Magnified views of box regions are shown in the upper-right panels.

As shown above, both liraglutide and GCGR mAb increased the *β*-cell area. Subsequently, we tried to determine the source of the increased islet cells. We performed costaining of insulin and PCNA in pancreatic sections to detect *β*-cell proliferation. We found that the proportions of PCNA^+^insulin^+^ cells (the proliferating *β*-cells) were higher in the liraglutide group and the mAb+Lira group when compared to the STZ group, while the proportion in the GCGR mAb group did not show differences with that in other groups (Figures 3(a) and 3(d), Table S1).

### 3.4. Liraglutide Induces Duct-Derived *β*-Cell Neogenesis in T1D Mice

Neogenesis from precursor cells is an important approach for the recovery of *β*-cell mass. Pancreatic precursor cells were often located near or within the adult pancreatic ducts [16]. Our previous study has proved that GCGR mAb could induce pancreatic duct-derived *α*-cell neogenesis, rather than *β*-cell neogenesis, in T1D mice [13]. In this study, we costained insulin or glucagon with pancreatic duct marker CK19 to evaluate the cell neogenesis. Results showed that in the liraglutide group or mAb+Lira group, glucagon-positive cells or insulin-positive cells could appear adjacent to CK19^+^ cells, suggestive of duct-derived *α*-cell or *β*-cell neogenesis (Figures 3(b) and 3(c)). However, the glucagon or insulin-positive cells that were located in the duct were rare, so we did not perform quantification further.

### 3.5. Liraglutide and GCGR mAb Treatments Induce *α*- to *β*-Cell Transdifferentiation in T1D Mice

**Figure 4:**
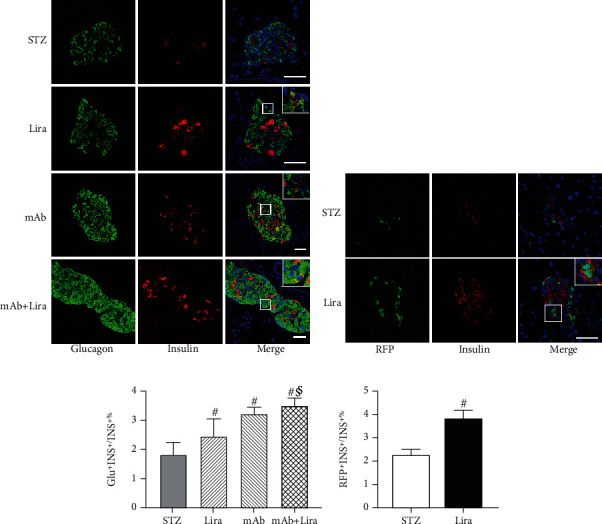
Pancreatic histological analysis of *α*- to *β*-cell transdifferentiation in T1D mice treated with liraglutide, GCGR mAb, or both for four weeks. (a) Representative images of insulin and glucagon colocalization. Scale bar = 50 *μ*m. (b) Representative images of insulin and tracing marker, red fluorescence protein (RFP), and colocalization in pancreatic *α*-cell tracing T1D mice. (c) Quantification of the glucagon+insulin+ cells. Scale bar = 50 *μ*m. (d) Quantification of the RFP+insulin+ cells. Data are expressed as the mean ± SEM. *n* = 6 − 9 sections/mouse multiplied by 6 mice/group. Statistical analysis was conducted by ANOVA or Student's *t*-test when appropriate. ^#^*p* < 0.05 vs. STZ group; ^§^*p* < 0.05 vs. liraglutide group. Magnified views of box regions are shown in the upper-right panels.

To evaluate *α*- to *β*-cell transdifferentiation, we performed glucagon and insulin double immunostaining. Compared with the STZ group, the proportion of glucagon^+^insulin^+^ cells were boosted by the liraglutide treatment (*p* = 0.031, Figures 4(a) and 4(c), Table S1). Next, we established pancreatic *α*-cell lineage-tracing (glucagonCre-RFP) mice. About 80% of glucagon-positive cells were RFP positive, suggestive of the high tracing efficiency, and there was almost no RFP^+^glucagon^−^ cells in the nondiabetic tracing mice, suggestive of no leakage of tracing (Figure S1). Therefore, RFP could be a good tracing marker of *α*-cells. After liraglutide treatment, the proportion of RFP^+^insulin^+^ cells was significantly higher than that in the STZ group (*p* < 0.01, Figures 4(b) and 4(d)). Notably, we not only found glucagon^+^RFP^+^insulin^+^ cells but also found glucagon^−^RFP^+^insulin^+^ cells; the latter suggested that the transformed *β*-cells lost glucagon expression (Figure S2). All the above results confirmed that some newborn *β*-cells were derived from the transdifferentiation of pancreatic *α*-cells.

GCGR mAb could promote *α*- to *β*-cell transdifferentiation, as indicated by the increased proportion of glucagon^+^insulin^+^ cells (*p* < 0.01), which was consistent with our previous report [13]. Notably, the proportion of glucagon^+^insulin^+^ cells in the mAb+Lira combination group was even higher than that in the liraglutide group (*p* = 0.038, Figure 4(c)).

### 3.6. The Regenerated *β*-Cells Own the Maturity Phenotype

**Figure 5:**
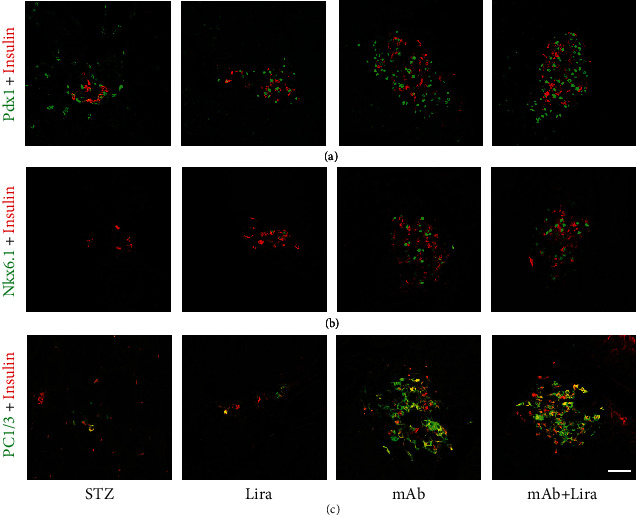
Pancreatic histological analysis of *β*-cell phenotype in T1D mice treated with liraglutide, GCGR mAb, or both for four weeks. (a) Representative images of insulin and Pdx1 (pancreatic and duodenal homeobox 1) colocalization. (b) Representative images of insulin and Nkx6.1 (NK6 homeobox 1) colocalization. (c) Representative images of insulin and PC1/3 (proprotein convertase 1/3), colocalization. Scale bar = 50 *μ*m.

To further investigate the identity of the neogenerated insulin-expressing cells above, thorough marker gene analyses were performed in all treated animals. Our data indicated that all insulin^+^ cells (including preexisting and neogenerated) uniformly expressed the bona fide *β*-cell labels, such as Pdx1, Nkx6.1, and PC1/3 (Figure 5). These analyses led us to conclude that the hyperplastic insulin-expressing cells observed after liraglutide or GCGR mAb treatment owned a *β*-like cell phenotype.

### 3.7. GCGR mAb Treatment Upregulates GLP-1 Receptor Expression

**Figure 6:**
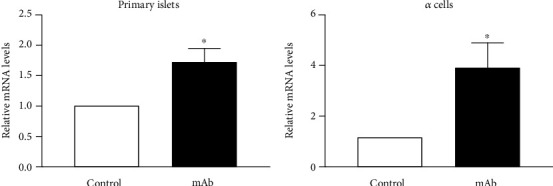
GLP-1 receptor expression induced by GCGR mAb treatment. The primary islets (a) and *α*-cell line *α*TC1.9 cells (b) were treated with GCGR mAb or IgG (as control) for 24 h, and GLP-1 receptor mRNA levels were determined by real-time RT-PCR. Statistical analysis was conducted by Student's t-test. ^∗^*p* < 0.05 vs. control group.

GLP-1 plays diversified effects mainly through the GLP-1 receptor [17]. To explore the potential mechanism of GCGR mAb synergistically promoting *α*- to *β*-cell conversion induced by liraglutide, we analyzed the levels of the GLP-1 receptor. In mouse primary islets and *α*-cell line *α*TC1.9, glucagon blockage by GCGR mAb upregulated the mRNA levels of the GLP-1 receptor (Figure 6).

## 4. Discussion

Our results demonstrated that the GLP-1 receptor agonist liraglutide increased pancreatic *β*-cell mass in T1D mice through self-replication, differentiation from precursor cells, and transdifferentiation of pancreatic *α*- to *β*-cells. Although combination of liraglutide and GCGR mAb did not demonstrate remarkable synergistic effects on the glucose level and *β*-cell area, the stimulating effects of GCGR mAb on the *α*-cell area and glucagon secretion were alleviated. Interestingly, transdifferentiation of pancreatic *α*- to *β*-cells was also boosted in the combination group. Increased GLP-1 receptor expression might be the possible reason of the synergetic effects of the two drugs. The combination strategy of the GLP-1 receptor activation with glucagon blockage may be beneficial in the T1D context, with good glucose control, *β*-cell regeneration, and not-very-high glucagon levels.

The current therapy for T1D is limited, and it is highly needed to evaluate the new nontraditional therapy for glucose control and maybe even for *β*-cell regeneration in T1D [18, 19]. GLP-1 receptor agonists have various beneficial effects on pancreatic *β*-cells, including promoting *β*-cell regeneration [20, 21]. However, most of the conclusions were obtained in T2D models. Our present study showed that a GLP-1 receptor agonist liraglutide increased pancreatic *β*-cell area in STZ-induced T1D mice. Moreover, we found that liraglutide not only promoted the proliferation of existing pancreatic *β*-cell and induced cells in the duct lining to transform into pancreatic islet cells, but also boosted *α*-cells to transdifferentiate into insulin-positive cells. In this way, we confirmed that all above sources participated in the liraglutide-induced *β*-cell renewal. However, liraglutide could not decrease blood glucose in T1D mice. The GLP-1-based therapy cannot be used alone for T1D treatment, and the combination with other drugs is needed.

Our previous study, together with others, has proven that GCGR blockage could decrease blood glucose and improve the phenotype of T1D mice. Strikingly, GCGR mAb increased the number of pancreatic *β*-cells and upregulated circulating insulin levels by inducing *α*- to *β*-cell transdifferentiation in T1D mice [13, 22]. However, GCGR mAb substantially increased pancreatic *α*-cell mass, which brings a safety concern on the *α*-cell tumor [23]. Notably, GLP-1 receptor agonists have the ability of inhibiting glucagon secretion and inducing *α*- to *β*-cell transdifferentiation. In this study, we tried to evaluate the synergistic effect of GCGR mAb and liraglutide in T1D mice. Although the combination did not show obvious advantages in decreasing blood glucose or increasing *β*-cell mass, the plasma glucagon level in the combination group decreased significantly and the *α*-cell area showed a downward trend, and notably, the *α*- to *β*-cell transdifferentiation was enhanced. Glucagon blockage by GCGR mAb could upregulate GLP-1 receptor expression, which might be the possible reason of the synergetic effects of GCGR mAb and liraglutide.

However, there were some limitations in our research. First, the immunostaining of PCNA with insulin, CK19 with insulin, and glucagon with insulin only displayed the proliferating, neogenetic, and transdifferentiated insulin-positive cells at a time point (before sacrifice). Although the low proportion at a real-time state might not contribute to the increased beta cell mass, the increased beta cells could be much more during the whole treatment period. Because we did evaluate the neogenetic cell proportion during the whole treatment period, so we could not conclude which source contributed most to the regenerated beta cells. Second, precursor-specific lineage tracing mice were needed for verification of stem cell-derived *β*-cells. Third, we only found that the mRNA expression of the GLP-1 receptor was upregulated by GCGR mAb, and its effects on the combination and other underlying molecular mechanisms need to be further confirmed.

In summary, our present study evaluated the synergic effect of GLP-1 receptor activation and GCGR antagonism in T1D mice. Although we did not find better glucose control and *β*-cell regeneration, we discovered that combination of liraglutide with GCGR mAb could promote *α*- to *β*-cell transdifferentiation, thus attenuating the GCGR mAb-induced *α*-cell hyperplasia and hyperglucagonemia. Our research may provide useful clues for the clinical therapy in T1D.

## Data Availability

The data used to support the findings of this study are available from the corresponding author upon reasonable request.

## Supplementary Materials

Supplementary Materials Figure S1: the efficiency of lineage-tracing. B6.Cg-Tg(Gcg-cre)1Herr/Mmnc and B6.Rosa26-LSL-Cas9-tdTomato/J mice were crossed to generate pancreatic *α*-cell lineage-tracing mice. Pancreas from eight-week-old mice was coimmunostained of glucagon and RFP. Figure S2: pancreatic histological analysis of *α*-to *β*-cell transdifferentiation in *α*-cell lineage-tracing T1D mice treated with liraglutide for four weeks. The arrowhead in the upper lane shows glucagon, RFP (the tracing marker of *α*-cells), and insulin colocalization. The arrowhead in the lower lane shows RFP and insulin colocalization with glucagon loss. Scale bar = 10 *μ*m. Table S1: quantification of plasma hormone and immunostaining in the pancreas.
