# Supplementary material for: Combination of GLP-1 Receptor Activation and Glucagon Blockage Promotes Pancreatic β-Cell Regeneration In Situ in Type 1 Diabetic Mice
Source: J Diabetes Res. 2021 Nov 25;2021:7765623. doi: 10.1155/2021/7765623 (PMC11401728; doi:10.1155/2021/7765623)
Supplement: Supplementary Materials — Figure S1: the efficiency of lineage-tracing. B6.Cg-Tg(Gcg-cre)1Herr/Mmnc and B6.Rosa26-LSL-Cas9-tdTomato/J mice were crossed to generate pancreatic α-cell lineage-tracing mice. Pancreas from eight-week-old mice was coimmunostained of glucagon and RFP. Figure S2: pancreatic histological analysis of α-to β-cell transdifferentiation in α-cell lineage-tracing T1D mice treated with liraglutide for four weeks. The arrowhead in the upper lane shows glucagon, RFP (the tracing marker of α-cells), and insulin colocalization. The arrowhead in the lower lane shows RFP and insulin colocalization with glucagon loss. Scale bar = 10 μm. Table S1: quantification of plasma hormone and immunostaining in the pancreas. [file 7765623.f1.docx]

##

## Supplementary materials for

## Combination of GLP-1 receptor activation and glucagon blockage promotes pancreatic β-cell regeneration in type 1 diabetic mice

**Liangbiao Gu, Dandan Wang, Xiaona Cui, Tianjiao Wei, Kun Yang, Jin Yang, Rui Wei*, Tianpei Hong**

**Supplementary figure legends**

**Figure S1 The efficiency of lineage-tracing.** B6.Cg-Tg(Gcg-cre)1Herr/Mmnc and B6.Rosa26-LSL-Cas9-tdTomato/J mice were crossed to generate pancreatic α-cell lineage-tracing mice. Pancreas from eight-week-old mice was co-immunostaining of glucagon and RFP.

**Figure S2. Pancreatic histological analysis of α-to β-cell trans-differentiation in α-cell lineage-tracing T1D mice treated with liraglutide for four weeks.** The arrowhead in the upper lane shows glucagon, RFP (the tracing marker of α-cells), and insulin co-localization. The arrowhead in the lower lane shows RFP and insulin co-localization with glucagon loss. Scale bar = 10 μm.

**Supplementary table**

**Table S1 Quantification of plasma hormone and immunostaining in the pancreas.**

| **Group** | **Control** | **STZ** | **Liraglutide** | **GCGR mAb** | **Lira+mAb** |
| --- | --- | --- | --- | --- | --- |
| glucagon (ng/mL) | 0.065 ± 0.0025 | 0.098 ± 0.0090 | 0.089 ± 0.0052 | 1.26 ± 0.16 | 0.60 ± 0.018 |
| insulin (ng/mL) | 0.71 ± 0.14 | 0.045 ± 0.012 | 0.071 ± 0.039 | 0.36 ± 0.080 | 0.38 ± 0.021 |
| islet area (mm^2^) | 0.084 ± 0.012 | 0.023 ± 0.0022 | 0.036 ± 0.0049 | 0.12 ± 0.017 | 0.10 ± 0.015 |
| β-cell area (mm^2^) | 0.075 ± 0.013 | 0.0038 ± 0.00031 | 0.0085 ± 0.0018 | 0.035 ± 0.0062 | 0.034 ± 0.0054 |
| α-cell area (mm^2^) | 0.0099 ± 0.0012 | 0.022 ± 0.0022 | 0.028 ± 0.0041 | 0.10 ± 0.016 | 0.065 ± 0.011 |
| β/α-cell area  proportion | 7.6 ± 1.5 | 0.17± 0.011 | 0.30 ± 0.042 | 0.35 ± 0.0043 | 0.52 ± 0.045 |
| PCNA^+^insulin^+^/  insulin^+^ (%) | - | 2.01 ± 0.30 | 3.16 ± 0.19 | 2.70 ± 0.31 | 3.18 ± 0.23 |
| glucagon^+^insulin^+^/  insulin^+^ (%) | - | 1.83 ± 0.15 | 2.44 ± 0.22 | 3.31± 0.22 | 3.44 ± 0.28 |
| RFP^+^insulin^+^/  insulin^+^ (%) | - | 2.33 ± 0.20 | 3.68 ± 0.26 | - | - |

Data are expressed as the mean ± SEM.
